# Impact of Postshock Transcutaneous Pacing on Chest Compression Quality during Resuscitation: A Simulation-Based Pilot Study

**DOI:** 10.1155/2021/5584632

**Published:** 2021-04-22

**Authors:** Wojciech Telec, Tomasz Kłosiewicz, Radosław Zalewski, Julia Żukowska-Karolak, Artur Baszko, Mateusz Puślecki

**Affiliations:** ^1^2nd Department of Cardiology, Poznan University of Medical Sciences, 149 28 Czerwca 1956r. Street, Poznan 61-485, Poland; ^2^Department of Medical Rescue, Poznan University of Medical Sciences, 7 Rokietnicka Street, Poznan 60-608, Poland; ^3^Students Scientific Circle of Medical Simulation, Department of Medical Rescue, Poznan University of Medical Sciences, 7 Rokietnicka Street, Poznan 60-608, Poland; ^4^Department of Cardiac Surgery and Transplantology, Poznan University of Medical Sciences, 1/2 Dluga Street, Poznan 61-848, Poland

## Abstract

**Background:**

Successful defibrillation is commonly followed by a transient nonperfusing state. To provide perfusion in this stagnant phase, chest compressions are recommended irrespective of arrhythmia termination. Implantable cardioverters-defibrillators (ICD) used immediately after delivery of the shock are capable of pacing the heart, and this feature is commonly activated in these devices. Potential utility of external, transcutaneous postshock pacing in patients with SCA in shockable rhythms has not been determined. This study aimed at presenting an impact of a short-term external postshock pacing (ePSP) on a quality of chest compressions (CC) without compromising them.

**Methods:**

The study was designed as a high-fidelity simulation study. Twenty triple-paramedic teams were invited. Participants were asked to take part in a 10-minute adult cardiac arrest scenario with ventricular fibrillation. In the first simulation, paramedics had to resume compressions after each shock (control group). In the second, simultaneous with compressions, one of the rescuers started transcutaneous pacing (TCP) with a current output of 200 mA and a pacer rate of 80 ppm. TCP was finished after 30 seconds (experimental group). The primary outcomes were chest compression fraction (CCF), mean depth and rate of compressions, percent of fully recoiled compressions, and percent of compressions of correct depth and their rate.

**Results:**

In both experimental and control group, CCF, mean depth, and rate were similar (84.65 ± 3.67 vs. 85.45 ± 4.95, *p*=0.54; 55.75 ± 3.40 vs. 55.25 ± 2.73, *p*=0.63; 122.70 ± 4.92 vs. 120.80 ± 6.00, *p*=0.25, respectively). In turn, percent of CC performed in correct depth, rate, and recoil was unsatisfactory in both groups (51.00 ± 17.40 vs. 52.60 ± 18.72, *p*=0.76; 122.70 ± 4.92 vs. 120.80 ± 6.00, *p*=0.25, respectively). Small differences were not statistically significant. Moreover, appropriate hand-positioning was observed more frequently in the control group, and this was the only significant difference (95.60 ± 5.32 vs. 99.30 ± 1.59, *p*=0.006).

**Conclusion:**

This difference was statistically significant (*p* < 0.01). Introducing an ePSP does not influence relevantly the quality of CC.

## 1. Introduction

Most patients after a successful defibrillation shock during routine advanced cardiovascular life support (ACLS) remain temporarily pulseless. Even if the defibrillation attempt is successful, it takes time until the postshock circulation is established. Severe bradycardia and complete heart block are frequently observed in the postshock period [1, 2]. Based on this phenomenon, the American Heart Association (AHA) recommendation is to resume chest compressions (CC) immediately after the shock delivery in all patients of unwitnessed cardiac arrest [3]. This protocol at least partially supports perfusion until the cardiac output becomes sufficient and pulse becomes palpable.

Interestingly, patients with implantable cardioverters-defibrillators (ICD) commonly undergo postshock pacing immediately after defibrillation. This is the feature that has been available for many years and has shown some benefits supporting the heart rhythm transiently after the successful shock [4]. There is a study reporting that short-term postshock pacing was required in as much as 40% of patients who underwent ICD shock delivery. The need for this pacing was proven in well-monitored anaesthesia units with cardiac output and mean arterial pressure monitoring [5]. This strategy of postshock pacing has not yet been explored for its use in adult ACLS situations of shockable cardiac rhythms.

External transcutaneous electrical stimulation of the heart has been introduced with promising results by Zoll in the mid-50s [6, 7]. Over following years, the method has been refined to overcome some technical issues, and a large number of studies were published, with repeating results—good response and improved outcomes in symptomatic bradycardia cases—and very poor results in patients with asystolic cardiac arrest [8–11]. Based on these findings, external pacing is recommended in symptomatic bradycardia, but not as a part of a routine asystole management.

It is important to notice that a majority of studies refer to external cardiac pacing in nonshockable rhythms. The utility of pacing following defibrillation immediately in cardiac arrest (postshock pacing) is virtually unexplored, and observations taken from nonshockable cases should not be translated into treatment of VF or pulseless VT. Potential utility of external, transcutaneous postshock pacing in patients with sudden cardiac arrest (SCA) has not yet been clearly determined. It seems possible that postshock bradyarrhythmias may respond to transcutaneous pacing, and to the best of authors' knowledge, there has been no attempt yet to create and test the algorithm of postshock pacing in VF/pulseless VT setting.

High-quality CC are an essential part of cardiopulmonary resuscitation (CPR). There is an undisputable amount of data showing that CC must not be interrupted if not absolutely necessary and the time with no compressions should be as short as possible [2]. Any additional procedure implemented into CPR must not negatively affect the quality of the compressions. The authors aimed at verifying the hypothesis that short-term external PSP (ePSP) could be delivered during resuscitation with no negative impact on the quality of CPR.

## 2. Methods

### 2.1. Legal Aspects

The study protocol was approved by the Institutional Review Board of Poznan University of Medical Sciences (no.: KB68/20). Written informed consent to participate in the study was obtained from all participants.

### 2.2. Participants

Twenty triple-person teams were invited to the study. The participants were paramedics working in ambulance teams, with at least three years of work experience. The participants were acquainted with the simulator in the scope of activities necessary to be performed during the research. Each participant completed a certified Advanced Cardiovascular Life Support (ACLS) course. In prebriefing, teams were instructed how to use the equipment and could practice each of the elements on the human simulator.

### 2.3. Study Design and Protocol

The research has been designed as a high-fidelity simulation study. Participants were asked to take part in a 10-minute adult cardiac arrest scenario with ventricular fibrillation. The participants were instructed to perform resuscitation according to the AHA ACLS algorithm. Each team performed two simulations. In between the two simulations, the paramedics rested for at least an hour.

**Figure 1:**
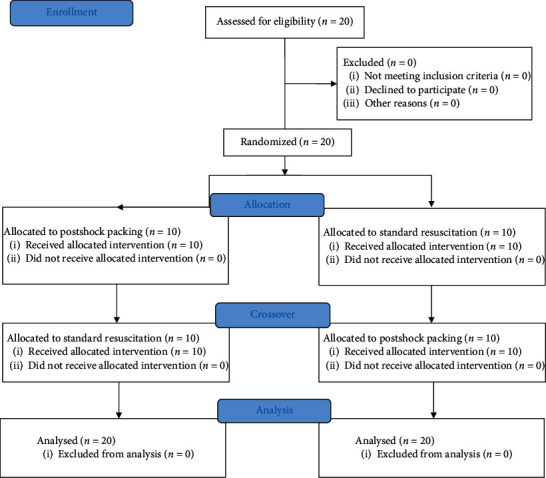
Study flowchart according to CONSORT statement.

In the first simulation, paramedics had to resume compressions after each shock (control group). In the second, simultaneous with compressions, one of the rescuers started transcutaneous pacing with a current output of 200 mA and a pacer rate of 80 ppm. The transcutaneous pacing was finished after 30 seconds from the onset (experimental group). Free online randomization tool was used to allocate the participants into the two groups (https://www.randomizer.org/). Time was measured with a stopwatch. The study protocol is shown in Figure 1.

### 2.4. Equipment

The ZOLL M Series monitor/defibrillator (ZOLL Medical Corporation, Chelmsford, Massachusetts, USA) was used to perform electrotherapy procedures. Either defibrillation or pacing was performed with the same self-adhesive pads.

The Resusci Anne Advanced Skill Trainer (Laerdal Medical AS, Stavanger, Norway) human simulator was used during the study. The simulator allowed generating the pulse on the carotid arteries, respiration, and heart rhythm. It was possible to perform defibrillation as well as TCP in real time using real energy. During the study, the parameters of chest compressions quality were measured with Session Viewer Software 6.2.6400 (SimVenture, South Newlands, United Kingdom).

### 2.5. Outcomes

The primary outcomes were (1) CC fraction (CCF), (2) mean depth, (3) rate of compressions, (4) percent of fully recoiled compressions, (5) percent of compressions of correct depth, and (6) their rate.

### 2.6. Statistical Analysis

The information was saved in Microsoft Excel 2010. Results were analysed in GraphPad Prism 8.0 (GraphPad Software Inc., San Diego, California, USA) and IBM SPSS and presented as means (SD). Differences between the two groups were determined using a two-tailed paired *t*-test and Wilcoxon test. A *P* value <0.05 was considered as significant.

## 3. Results

The analysis of the records revealed that the mean depth of compressions in both groups was within the normal range. The rate of compressions was slightly faster in the experimental group, but this difference was not statistically significant.

The percentage of compressions performed at the correct rate, and the appropriate recoil, was unsatisfactory in both groups. Small differences between the two groups were statistically insignificant.

Proper hand positioning was more often observed in the control group (99.30% vs. 95.60%), and this difference was statistically significant (*P*=0.006). The detailed results are summarized in Table 1.

## 4. Discussion

According to the best of the author's knowledge, this simulation research is pioneer to study the feasibility of using ePSP in resuscitation with shockable rhythms.

Currently, it is not recommended to perform any additional interventions other than chest compressions after the shock delivery [3]. Several studies have shown no benefit of early transcutaneous pacing in patients who experienced out-of-hospital asystolic arrest [12]. Contrarily, Hazard et al. have proved the effectiveness of transvenous cardiac pacing as a method of ceasing acute bradycardia during CPR [13]. Postshock pacing programme in ICDs served as an inspiration for this research. ICD programs, depending on the manufacturers, and enabled automatic 30-second heart stimulation immediately after shock. Clinical analyses in this subject have proved it effective to cease various bradyarrhythmias that occur during shock [14].

When analysing experience with implantable devices, it can be assumed that such an intervention may also be effective when external pacing devices are used. The main generally-known weakness is a difficulty with obtaining good ventricle capture of the stimuli. Furthermore, pacing thresholds may change without warning and capture can readily be lost. Benton found that, of patients in whom complete heart block or other bradycardia occurred during CPR, capture was possible in 77% [13].

The aim of correct CC is to maintain the perfusion of brain tissue and proper coronary perfusion pressure. High-quality CC increase the chances of survival by 1.5–4 times. The parameters of CC quality are of correct depth (5-6 cm), at a correct rate (100–120/min), full chest recoil. Interruptions in compressions must also be minimized. It is recommended to resume CC immediately after defibrillation and continue for the next 2 minutes or until the return of spontaneous circulation is observable [3]. Higher CCF has also been shown to correlate with higher survival and better neurological outcomes. It can be achieved when CCF amounts to 81% and more [15]. The best quality parameters for CC are achieved when using automatic CC devices (ACCD). However, ACCD is not currently recommended for routine use [16].

So far, CC have been one of the few activities whose impact on the survival of patients with SCA has been thoroughly proven. Therefore, it is important that this procedure is carried out in high-quality during each resuscitation. So far, it has not been proved that ePSP improves patients' survival. The studies on this subject are out-of-date and mostly come from the 80's of the last century. Since then, the guidelines for resuscitation have been modified many times. Furthermore, percutaneous stimulation devices are available for prehospital care. This procedure may be performed by paramedics, and no physician attendance is needed. Operation of these devices is simple. The same adhesive electrodes are used for the procedure as for defibrillation. It was proven that the use of multifunction electrodes is more efficient in comparison with standard paddles. The former improves the quality of cardiopulmonary resuscitation run by a two-paramedic team [17].

There are no additional costs to performing the procedure, assuming the defibrillator has a pacing feature. In the authors' opinion, it is worth to highlight the possibilities of using this procedure and perform additional studies that could evaluate its real effectiveness in modern medicine.

## 5. Limitations

The authors are aware of the limitations of this study. The study was performed under simulation conditions. This environment allows, however, to check the quality of chest compressions without interfering with the patient's safety. The presented study confirms that ePSP does not have a negative impact on CPR, but does not respond to question of whether ePSP improves CPR outcomes. The authors' intention was to demonstrate the possibility of using the procedure without affecting the quality of CPR. Such a study has not been performed so far nor presented in paper results, what confirms that the subject of ePSP concept should be developed. The use of ePSP in ACLS may be reasonable. However, this procedure should not be used if it would negatively affect actions with well-known effectiveness. So far, only chest compression between defibrillations is the best option for improving no-flow time right after shock.

This study is the beginning of the authors' work on ePSP. Further long-term observational studies are needed.

## 6. Conclusions

According to the results of this study it can be concluded that the use of ePSP does not affect negatively the quality of performed CC.

## Figures and Tables

**Table 1:** The detailed results of the research.

a	Experimental group (postshock pacing) *n* = 20	Control group *n* = 20	*P* value
CCF (%)	84.65 ± 3.67	85.45 ± 4.95	0.54
Correct hand placement (%)	95.60 ± 5.32	99.30 ± 1.59	0.006
Mean number of compressions per single scenario	1068 ± 46	1077 ± 43	0.50
Mean depth (mm)	55.75 ± 3.40	55.25 ± 2.73	0.63
Correct depth (%)	51.00 ± 17.40	52.60 ± 18.72	0.76
Correct recoil (%)	35.35 ± 20.69	40.50 ± 22.28	0.43
Mean rate (1/min)	122.70 ± 4.92	120.80 ± 6.00	0.25
Correct rate (%)	39.40 ± 20.21	42.45 ± 22.40	0.45

^a^Continuous variables are presented as means ± SD. CCF: chest compression fraction.

## Data Availability

The data are available from the authors upon reasonable request.
